# Moral Enhancement Should Target Self-Interest and Cognitive Capacity

**DOI:** 10.1007/s12152-017-9331-x

**Published:** 2017-04-26

**Authors:** Rafael Ahlskog

**Affiliations:** Department of Government, Uppsala Universitet, Uppsala, Sweden

**Keywords:** Moral enhancement, Cognitive enhancement, Self-interest, Sense of self, Psychedelics

## Abstract

Current suggestions for capacities that should be targeted for moral enhancement has centered on traits like empathy, fairness or aggression. The literature, however, lacks a proper model for understanding the interplay and complexity of moral capacities, which limits the practicability of proposed interventions. In this paper, I integrate some existing knowledge on the nature of human moral behavior and present a formal model of prosocial motivation. The model provides two important results regarding the most friction-free route to moral enhancement. First, we should consider decreasing self-interested motivation rather than increasing prosociality directly. Second, this should be complemented with cognitive enhancement. These suggestions are tested against existing and emerging evidence on cognitive capacity, mindfulness meditation and the effects of psychedelic drugs and are found to have sufficient grounding for further theoretical and empirical exploration. Furthermore, moral effects of the latter two are hypothesized to result from a diminished sense of self with subsequent reductions in self-interest.

## Introduction

Do humans possess a large enough capacity for morality to safely navigate the minefield of modern, high-tech civilization [1]? If not, would it be admissible to enhance moral capacities or moral behavior and thereby avoid certain catastrophic calamities? How would one go about doing so, and what traits ought to be targeted? The moral enhancement debate has covered these questions extensively in recent years, with a large variety of viewpoints regarding the necessity, admissibility and practicability of different supposed moral enhancement interventions. Furthermore, a number of different capacities have been suggested as potential targets – aggression, empathy, self-control etc, as well as a number of specific biomedical interventions [2].

In this paper I will argue, based on a novel formal model of moral capacities, in favor of a previously underappreciated route to moral enhancement: rather than *increasing* certain moral or prosocial motivations directly (which as we shall see is likely to have significant unintended consequences), a more reliable path would be to *diminish* self-interested motivation, thereby allowing the moral sentiments that people already possess naturally more room to flourish. Further, I argue that this can be synergetically supplemented with cognitive enhancement. My argument is thus partially in line with both Earp et al. [3], who argue that in certain cases, diminishing a capacity can actually be an enhancement, and with authors like Harris [4], Carter & Gordon [5] and Earp et al [6], who in the context of moral enhancement argue in favor of second-order moral capacities such as reasoning.

In the following pages, I will first briefly go through the different definitions of moral enhancement, as well as the types of candidate interventions, that have previously been put forth. In so doing, I will also position my argument among the many possible taxonomies of enhancements that have been outlined by others. I then move on to sketch a simple unified model of prosocial motivation that summarizes some fundamental dynamics of how at least *some* of our moral sentiments likely function. This model provides the two key insights presented above, and also suggests an important argument for why moral enhancement specifically targeting our capacity for empathy may not be an ideal route and would likely backfire. Finally, I will argue that my proposals are well in line with a large body of empirical research on, for example, mindfulness meditation, psychedelic drugs, and cognitive enhancements, and warrant further theoretical and empirical consideration.

The contribution of this paper is thus two-fold. To begin with, I suggest a new way of viewing moral decision making in the context of the enhancement debate formally as a type of constrained optimization problem. Second, the paper contributes to the discussion of which capacities that ought to be, or ought not to be, targeted for moral enhancement.

## What do I Mean by Moral Enhancement?

What one means by a specifically *moral* enhancement is, first of all, naturally highly dependent on one’s ethical foundations. Here, a sort of “lowest common denominator” approach is assumed. It should be clear that most ethical systems rely, at least to some extent, on the ability of moral agents to take the welfare or interests of others into account.^1^ How it should be aggregated, how different others should be weighed against each other, and ultimately how this ability should guide behavior can, has been and will be subject to (possibly intractable) debate. At the very least, however, the capacity for other-regard seems to be a necessary component of almost any wide-spread conception of morality. By this minimalist approach, a moral enhancement intervention is any intervention that would make an agent more likely to consider someone else’s welfare in a given situation, without making it less likely in another situation or toward another agent (the Pareto principle). By someone else, I also here mean any person that is not oneself, be they close family or distant strangers. Morality, using this approach, is then by necessity at least to some degree “prosocial” in the broad sense of the word.

Defining what constitutes moral *enhancement* is itself a fraught enterprise, and different suggestions abound (see Raus et al [9] for an overview). A fundamental distinction is that between treatment and enhancement, where treatment would be bringing a person of substantially less than average moral capacities up to average, and enhancement would be bringing the moral capacities of an agent to a level above average. This paper is concerned only with enhancement in a strict sense: my argument, crucially, does in fact not apply to what can be defined as a morally clinical population (psychopaths or people with other anti-social disorders). Rather, I assume a population of non-clinical, “normal”, or moderately prosocial agents.

Defining enhancement in this context is also a matter of pinning down precisely what problem it is intended to solve. A central argument from Persson & Savulescu [1] and others is on avoiding so called Ultimate Harm – a disaster so consequential that life would no longer be worth living on the planet. The risk of such a disaster, it is argued, is rapidly increasing as humans acquire technological means, vastly surpassing those of our evolutionary past, to cause changes to our current natural and social environment. It is worth distinguishing between different reasons for *why* this can lead to Ultimate Harm. On the one hand, a few morally corrupt individuals may soon face only small practical obstacles to creating weapons of mass destruction that put the entire world in peril. In accordance with the strict enhancement, rather than treatment, perspective, my suggestions for possible routes to moral enhancement cannot in fact solve this problem. Other interventions are required for this category of individuals.

On the other hand, there are possible Ultimate Harm (or perhaps, at the very least, severe harm) scenarios resulting from collective action problems posed by the aggregate outcomes of small-scale individual, and narrowly self-interested, decisions (the problem comes with many names: collective action, public goods dilemmas, the tragedy of the commons, and is discussed at length by Persson & Savulescu in chapter 6 [1]). Climate change, antibiotic resistance – and when focusing on larger actors, arms races – are such examples. These failures of collective action result when the individual contribution to the problem is not large enough to motivate abstaining from a particular action, but the end result of most people (or states) acting on their self-interest is an equilibrium substantially below what is socially optimal. In terms of risks of Ultimate Harm, collective action dilemmas, rather, is the category of problems that I aim for in this paper.

Another proposed taxonomy is on external vs. internal enhancements – that is, external means of controlling behavior, such as nudges, policing etc on the one hand, and direct modulations of neural systems that *essentially* change the agent on the other (Danaher [10] who further suggests that internal means of enhancement may be politically preferable and should be prioritised). My suggestions all fall in the internal category and are therefore in line with Danahers argument on preferability.

A third problem is to distinguish which particular traits or emotions that ought to be targeted by moral enhancement interventions, such as aggression, capacity for empathy, sense of fairness or self-control [2, 11, 12]. Another distinction is proposed by Earp et al [6], namely between first-order and second-order moral capacities. The authors argue that targeting first-order moral capacities, defined as more basic features of our moral psychology (which would include traits like empathy), is more likely to result in significant unintended consequences than targeting second-order ditto, that is, capacities that allow an agent to *regulate* the first-order capacities (reasoning and self-control would be examples of this).

Lastly, specific interventions for modulating these traits have also been suggested, such as oxytocin, serotonin or SSRI’s, *β*-blockers and psychedelic drugs such as psilocybin [13, 14]. In this vein, I shall return to the topic of psychedelics in detail later.

## A Model of Prosocial Motivation

Recorded history shows us that humans are capable of an extremely wide range of social behavior. Consequently, research on human prosociality has shown it to vary along several dimensions. We are more likely to behave altruistically towards kin than non-kin [15], towards people at a closer social proximity [16] and also towards those we can specifically identify [17]. These dimensions are likely consequences of several types of evolved prosocial reflexes, for example kin altruism [18] and reciprocal altruism [19]. Whereas the first conferred evolutionary benefits through increasing the reproductive chances of genetically related individuals, the latter functioned as a form of primitive insurance scheme in times of fluctuating food availability.

I hypothesize that the clear imbalances over possible subjects of prosocial behavior, and the existence of different evolutionary rationale for such, also suggest the likely existence of different proximal psychological mechanisms. I therefore propose that we can fruitfully analytically distinguish between three basic forms of motivation. First, we can distinguish between self-interested and other-regarding motivation,^2^ and then the latter into two distinct and qualitatively different types, namely *narrow* and *wide* other-regard. Self-interest here refers to self-interest in the narrowest form: increasing one’s own welfare, excluding effects on one’s own welfare that are mediated by effects on others.

Narrow other-regard concerns our closest circle of kin and friends – that is, they are both identifiable and at close social proximity. Narrow other-regard is likely at least partially driven by emotional empathy, possibly involving mirror neurons (emotional empathy in fact probably evolved from child-rearing in the first place – see for example Decety [20]).^3^

Wide other-regard concerns all those we perhaps cannot directly observe, who are at a long social distance or who might be very different from us (that is, out-group). Wide other-regard also captures all of those issues that perhaps do not concern any particular person or people, but rather society (local or global) as such. Environmental issues such as climate change fit here. This type of motivation may be more likely to be driven by cognitive empathy or perspective taking (or in the case of reciprocal altruism, by deservingness).

Further, the individual profile of self-interest and narrow and wide other-regard is far from static. On the contrary, it can vary over time, between decision frames and, most importantly, as life circumstances change.

A diverse range of evidence support the differing logic between the two types of other-regard. To begin with, the peptide hormone oxytocin is suspected to be intimately involved in human sociality. It is secreted during childbirth and in response to touch, and appears to increase cooperation in various game theoretic settings. This effect, however, seems to be limited to in-group individuals. Towards out-group individuals, it rather appears to produce increased spite [23]. If these effects are real,^4^ it underscores that increases in some form other-regard can result in decreases in other forms – reinforcing that caring more for close ones may not at all imply caring more in general, but may in fact *decrease* caring for a wider group.

Additionally, the drug MDMA (often known under the name Ecstasy) is well-known for producing massive surges in emotional empathy. This effect has been established under controlled circumstances [24], but again, seems limited to the narrow group: empathy is increased towards friends but not towards strangers [25]. This is also consistent with the theorized qualitative difference between narrow and wide other-regard.

From a developmental psychology perspective, it has been shown that in-group altruism develops at about the same time as out-group spite: they appear to be two sides of the same coin [26]. Even from an evolutionary perspective, it has been proposed that altruism and parochialism must have coevolved – altruism could not have arisen without the other side of the coin [27]. This, again, validates the difference between other-regard directed at a wide and a narrow group, and also shows that narrow and wide other-regarding behavior are not *independent* capacities.

It has also been shown that when humans have offspring, our nepotistic tendencies are heavily accentuated: when becoming a parent, the wider group becomes less important and one tends to discriminate more based on kin [28].

On the basis of the above cited evidence, I can construct a simple model of human prosociality that summarizes how the different motivations relate to each other and to behavior (and consequently how different types of behavior relate to each other). For the formally inclined: the model is designed as a constrained optimization problem whereby an individual allocates different amounts of resources to different people depending on the magnitudes of her different prosocial sentiments. Although the mathematics are laid out in detail in the Appendix, I will be referencing the following quantities throughout the text: *G* is some good (a particular resource, amount of cognitive energy or effort that an individual is capable of, or simply time spent awake) that is to be distributed between the self, the narrow group and the wide group, with the quantities for each denoted *g*_*s*_, *g*_*n*_ and *g*_*w*_. Furthermore, the distribution of the good *G* is decided on by each individual based on the magnitude of self-interest, narrow other-regard and wide other-regard, denoted *s*, *n* and *w*, respectively.

The proposed model, albeit crude, carries a simple but empirically viable logic regarding the interdependence of different moral capacities. The essential dynamic derived from the model is that just like everything else, moral capacities follow a form of economy. Since effort, goods and time are limited resources, one cannot easily change one moral sentiment (more specifically in this case, type of other-regard) without *also* affecting behavior towards subjects not directly targeted by that sentiment. A metaphor for this could be money spent on charity (symbolizing the wide group): with a given income to spend, if one were to increase the moral sentiment to give to charity, the amount of money spent on oneself and one’s family (the narrow group) has to decrease by necessity. In the terminology of the model: if wide other-regard *w* increases, the amount of the good *G* that is allocated to *g*_*s*_ and *g*_*n*_ decreases.

The implications of this are that if we modulate emotions that drive narrow other-regarding *motivation* (such as emotional empathy), this will have adverse effects on prosocial *behavior* directed at the wider circle – even when the wider moral sentiment remains unchanged! Similarly, if we increase regard for the wider circle, less resources would be spent on the narrow group whether this was intended or not.

## Proposals for Moral Enhancement

As we shall see, there are two simple ways out of the dilemma posed by the necessary interdependence of moral motivations, if moral enhancement is indeed what we are after. Below, I dig into these two complementary routes, and highlight existing and emerging empirical evidence that appears to fundamentally support the mechanisms I propose. It’s important, however, to keep in mind that the following discussion does not apply to the special case of antisocial preferences or psychopathy. The implications derived here do not apply when there is no preexisting (however small) level of prosocial motivation. These special cases, rather, should perhaps be handled as a clinical problem in its own right, and subjected to its own set of interventions not covered here.

### Target Self-Interest

The first implication for moral enhancement concerns what prosocial motivation it ought to target. As we have seen, we can derive from the model that targeting other-regard directly (through capacity for empathy or other traits), is likely to produce unintended consequences for the complementary other-regarding motivation. When emotional empathy or narrow other-regard is targeted for moral enhancement, we can expect this to not only increase narrow prosocial behavior, but also *decrease* wide prosocial behavior. Similarly, targeting cognitive empathy or wide other-regard may well lead to decreased narrow prosocial behavior.^5^ Mathematically speaking, there is a simple solution to this conundrum: target self-interest. Thus, a possible avenue for successful moral enhancement interventions may lie in *decreasing* a motivation, rather than increasing another. When self-interest *s* is decreased, *both* types of prosocial behavior (*g*_*n*_ and *g*_*w*_) increase.^6^

Another way of viewing the same dynamic is that by at least partially removing the constraint of self-interest, there will be more room for already existing prosocial sentiments in any given decision. Returning to the metaphor of money: with a given amount of money to spend on oneself, one’s family (the narrow group) and charity (the wider group), what is the only way to make sure that both the narrow and the wide others get a larger share? To decrease the motivation to spend it on oneself.

How does one go about decreasing self-interest, if not through the route of increasing other-regard? I shall here argue that, although analytically distinct, there is a natural connection between self-interest as a motivation, and *sense of self*. I here use sense of self not in the meaning of self-esteem, self-worth or self-knowledge, but to mean the rigidity with which one identifies as an independent entity from the rest of the world. To elaborate on the concept used in this way, and explore its links with morality, I will take a few examples from existing research.

One pertinent example comes from research into the prosocial effects of exposure to overwhelming or beautiful sensory stimuli. Piff et al [32] found in a set of experiments that inducing a sense of awe, for example by means of exposure to grand natural scenery, produced a diminished sense of self, and subsequently increases in prosocial behavior. This, thus, is in accordance with this prediction of the model proposed above: a diminished sense of self resulted in decreased self-interest *s*, which produced increases in the complementary goods. In the words of the authors: “reduced sense of self may allow people to transcend self-interest and behave in accordance with their higher moral values” (p. 896).

There is also evidence to suggest that the practice of mindfulness meditation may essentially display the same process: decreased sense of self, followed by increased other-regarding behavior.^7^ In their review of existing evidence on mindfulness, Hölzel et al [33] argue that changes in the perspective on one’s self is one of the mechanisms that explain the effects of the practice. More specifically, mindfulness meditation may allow the practitioner to diminish the sense of self and cause a shift toward “a more self-detached and objective analysis of … sensory events” [34]. Research into neural correlates of these phenomena also supports a diminished sense of self as a mechanism for their effects (see for example Lehman et al [35]).

Connecting these changes in sense of self to moral behavior, several studies have shown mindfulness practice to increase subsequent prosociality. For example, two studies have found that subjects who received mindfulness practice (8 weeks with a teacher in Condon et al [36] or 3 weeks with an online training application in Lim et al [37]) were more likely to give up their chair to a needy confederate than were subjects in control groups.^8^ Though these prosocial effects may well turn out to be mediated by other mechanisms than a decreased sense of self, they are consistent with such an explanation.

Another possible intervention that achieves a diminished sense of self has been present in the literature for some time, but appears to have yet to make it to the fore of the moral enhancement debate – namely psychedelic drugs. In his paper “Moral Transhumanism: The Next Step”, Michael Tennison [13] argues that psilocybin, the active component in many different species of psychedelic mushroom, is a potentially viable, safe and effective moral enhancement intervention. More recently, Earp et al [6] give several examples of how naturally occuring psychedelic substances have been used in shamanic settings for hundreds of years as a form of catalyst for moral learning.

Psychedelics such as psilocybin, LSD and DMT are well-known to cause a substantially diminished sense of self in higher doses.^9^ Recent studies have linked this phenomenon to specific neural correlates involved in self-awareness [40–42] which shows that, at least temporarily, the decreased sense of self that is often deliberately sought in this context may occur in a quite literal sense, rather than as a by-product of for example increases in empathy.^10^

Further, substantial experimental research has found that psilocybin, administered by clinical staff under carefully controlled and supportive circumstances, reliably causes sustained increases in the personality domain of openness [44], as well as moderate to extreme positive behavior changes, such as increases in altruism and decreases in judgmental attitudes, both immediately following the session as well as fourteen months later [45–47]. It has also been shown that use of psychedelic drugs among convicted offenders is associated with reduced recidivism, whereas other drug use is associated with the opposite [48], and that LSD produces acute increases in pro-social sharing behavior in an SVO task [43].

It thus appears that on a theoretical level, decreasing self-interest (for example via sense of self) may be a more friction-free route to moral enhancement, and that interventions utilizing, for example, psychedelic drugs, may show pro-mise in achieving this.

### Cognitive Enhancement can be Moral Enhancement

The second implication of the model is the following: if some level of prosocial motivation is present, and preferably a decrease in self-interest has already been achieved, certain types of cognitive enhancement would further increase the amount of energy spent on increasing the welfare of (all) others, and therefore the quality of prosocial behavior. This implication addresses the sum component *G*, that is, the sum of available resources that one can spend. One way of interpreting the limiting resource *G* is, as mentioned, in terms of available mental energy. That is, how much effort one can spend on increasing the welfare of oneself or others is limited. The implication is that if this limited resource could be increased, provided some level of prosociality is already existing, the distribution of good (or in this case effort) on all three components would increase – including the wide and the narrow group.

This argument has previously been made in the literature, although in slightly different forms. First of all, John Harris [4] famously argues, contra Persson & Savulescu, that stupidity is at least as dangerous as malice, and that cognitive enhancement would therefore on the balance decrease the risk of Ultimate Harm. Further, Carter & Gordon [5] argue that moral enhancement probably must involve some form of cognitive enhancement, since moral reasoning is itself a form of reasoning.^11^ Finally, Earp et al [6] touch on a similar argument when they propose second-order, rather than first-order, moral capacities to be a superior target for moral enhancement.

The argument I propose for cognitive enhancement as a form of moral enhancement is related but distinctly different. Rather than focusing on the danger of stupidity or the complexity of moral reasoning, my argument hinges on preexisting moral sentiments (*n* and *w* in the model), and cognition as a resource to be distributed on increasing the welfare of self and others. When there is more of the resource, any level of moral sentiment will make sure that there are at least some increases in cognitive resources spent on others than oneself. Thus, while an increase in *G* would also benefit the self (*g*_*s*_), it is, in economic terms, a Pareto improvement: nobody loses. Building on the previously mentioned metaphor of money spent on charity: a poor man can spend very little, despite possessing moral sentiments, but after suddenly getting a high income, he can safely spend more on charity even when his moral sentiments are untouched.

Given that there is very limited research into the effects of existing cognitive enhancers on prosocial behavior, there are two lines of empirical evidence that could conceivably inform this discussion. First, research on the effects of fatigue and sleep deprivation on prosocial behavior could illuminate effects of lowering *G*. Second, the relationship between general cognitive ability or IQ and prosocial behavior could say something about people with higher *G*.

While the existing evidence can in fact be interpreted as being consistent with the proposed mechanism, it is quite tricky to disentangle, for three central reasons. First, ceteris paribus conditions are hard to meet. That is, both fatigue and general cognitive capacity are likely *also* related to other variables in the model. For example, sleep deprivation is known to dampen empathy [49]. This means that any detrimental measured effects on prosocial behavior might be either due to a lower *G* *or* due to lowered *n* or *w*, which rules out definitive conclusions about my proposed mechanism. Similarly, high cognitive capacity may itself be correlated with moral sentiments, which thus confounds any relationship with moral behavior transmitted through cognitive resources spent on others.

Second, many studies on prosocial behavior utilize economic game experiments (both Anderson & Dickinson [50] and Ferrara et al [51] for example, look at the effects of sleep deprivation on economic game performance). Here, the limiting factor is no longer cognitive capacity to be distributed but rather an actual allotment of resources (money) to be distributed in for example a dictator game. Thus *G* is, in this sense, constant regardless of mental energy.

Third, as stated above, my argument hinges on high enough preexisting levels of *n* and *w* (and/or low enough levels of *s*) for changes in *G* to have the desired effect.^12^ To empirically measure the theorized effect, we thus need to test the *combination* of moral and cognitive enhancement.

The evidence on IQ and prosociality also appears somewhat mixed. Some studies have found modest to moderate positive correlations, while others have found none (Han et al [52] provides a comprehensive overview). Crucially, Bar-Tal et al [53] found that higher intelligence was associated with higher *quality* rather than higher quantity prosocial behavior, which is precisely what the model would predict.

Thus, while there is certain evidence that cognitive resources are somehow related to moral behavior, and consequently *might* be a form of moral asset in its own right, the available evidence is largely insufficient to know whether this is *also* partly due to the proposed mechanism.

## Discussion

In this paper I have laid out a simple model of how different types of motivations relate to prosocial behavior directed at different target groups. While the model can easily be expanded to encompass more, and more complex, types of moral motivations and behaviors, the basic dynamic remains: when it comes to viable moral enhancement for the general population, the most efficient route is not likely through modulating prosocial motivation or empathy at all, but rather self-interest.^13^ Different strands of evidence, such as on the effects of overwhelming sensory stimuli, mindfulness meditation, as well as psychedelic drugs, are all consistent with the idea that decreasing the self-interest (in these cases, through diminished sense of self – or “weakening” the ego, if you will) can have measurable effects on moral behavior.

This argument contrasts to some proposals about empathy enhancement that regard empathic circles rather than the “strength” of empathic capability (see for example Jebari [11]). Such proposals are similarily based on the idea that at least certain types of empathy might be limited to people within a particular circle (comparable to the notion of narrow other-regard), and that moral enhancement should aim at expanding this circle. This is a compelling proposal, but suffers from a number of weaknesses that, analytically speaking, intervening in self-interest doesn’t have.

One objection is that of empathic fatigue: the more people we empathize with, and the more intensely we empathize with them, the faster we will tend to become emotionally drained and experience a form of emotional burnout. This phenomenon has been documented in many professions where one is exposed to large amounts of suffering of others – for example, counseling [54], social work [55] and nursing [56]. It is also not clear that there are yet any feasible options for how to accomplish an empathic expansion, other than traditional means of exposure to a wider range of human or non-human subjects (like literature, travel etc), that has so far indeed managed to cause great expansions of our empathic circles – but that nonetheless appear insufficient in our present condition. Last but not least, if the model outlined in this paper carries water, interventions aimed at decreasing self-interest will increase prosocial behavior *regardless* of whether the recipient is inside or outside of our empathic circle and therefore covers strictly *at least* all the cases of prosocial behavior that would be induced by enlargement of empathic circles.

A second implication of the model is that cognitive enhancement, to the extent that it leads to additional cognitive resources to be expended on increasing the welfare of any recipient (whether self or others), can also lead to more prosocial behavior. While the connecting empirical evidence is merely weakly consistent with this proposal (in the sense that any direct tests of the suggested mechanism are lacking), it is nonetheless a theoretically well-grounded conclusion.

Whether the changes in moral behavior that are hypothesized to result from a decrease in self-interest and an increase in cognitive capacity are actually moral *improvements* is as mentioned a question at least partially dependent on what ethical system they are evaluated against. It should nonetheless be clear that, if I am correct, they do solve some of the fundamental issues put forward by proponents of moral bioenhancement. They do not, however, solve all problems. The largest remaining problem is with potential clinical populations: none of these hypothesized effects are applicable to people that, at the outset, lack any moral sentiments. Thus, they do not solve the moral issues underlying antisocial tendencies or psychopathy. These problems will have to be subjected to other interventions.

The ethical issues involved in meddling with self-interest, by pharmaceutical interventions or otherwise, have also not been explored in this paper. It would appear that, at least to some extent, it carries significantly different ethical problems than that of cognitive enhancement. Whereas cognitive enhancement, even when leading to more prosocial behavior, is on the face of it an individual good, it is not as clear that decreases in self-interest is an individual good so much as it is a collective good. There may well be cases in which it is an individual bad – at the extreme low end of self-interest is self-sacrifice. The extent to which such situations arise is likely to be a function of just how much self-interest remains after a successful moral enhancement intervention.

Empirical evidence can only go so far before actual moral or cognitive enhancement interventions are available. However, as such interventions are developed (if they are), and should it prove ethical to do so, I propose that a combination of decreased self-interest and increased cognitive capacity should be the prime targets when considering moral enhancement. Whether this, or any other type of moral enhancement, turns out to work remains to be seen.

## Appendix

Let’s begin with a utility function over the distribution of some good *G* between self (with weight *s*), the narrow group (with weight *n*) and the wide group (with weight *w*). These weights represent the forces of self-interest, narrow other-regard and wide other-regard, respectively. For simplicity the utility function *u* for the individual *i* has the following additive form (although crucially, the fundamental dynamics of the model are the same even with more complex specifications):

1 $$ u_{i}=s \sqrt{g_{s}}+n \sqrt{g_{n}}+w \sqrt{g_{w}}  $$

with the maximization restriction

2 $$ G=g_{s}+g_{n}+g_{w}.  $$

Note that the utility function exhibits diminishing marginal utility in each of the components (square roots of each). This makes substantive sense and also prevents corner solutions. Maximizing *u*_*i*_ subject to the restriction *G*, using Lagrangian multipliers, I can obtain the utility maximizing distribution of *g*_*s*_, *g*_*n*_ and *g*_*w*_ as functions of the weights *s*, *n* and *w*:

3 $$ g_{s}=\frac{s^{2}}{s^{2}+n^{2}+w^{2}},  $$

4 $$ g_{n}=\frac{n^{2}}{s^{2}+n^{2}+w^{2}},  $$

5 $$ g_{w}=\frac{w^{2}}{s^{2}+n^{2}+w^{2}}.  $$

As is evident from these results, each *g* is decreasing in both of the complementary weights and increasing in its own weight. That is, increasing any of the three weights would decrease distribution of good to the other two components. Consider for example how the wide good *g*_*w*_ changes in response to alterations to the three weights:

6 $$ \frac{\partial g_{w}}{\partial s}= -2sw^{2}G,  $$

7 $$ \frac{\partial g_{w}}{\partial n}= -2nw^{2}G,  $$

8 $$ \frac{\partial g_{w}}{\partial w}= 2wG(n^{2}+s^{2}).  $$

We can see that if self-interest *s* is increased, wide good decreases (6). As does it when narrow other-regard *n* increases (7). Wide good increases only with wide other-regard *w* (8). This result is symmetrical for the other two portions of good (self and narrow). That is, whenever one motivation is increased, the other two portions of good necessarily decrease.

## Footnotes

1 The Golden Rule, commonly codified in many of the major world religions, explicitly requires consideration of others. Both contractualism and contractarianism, meanwhile, require the consideration of other’s interests, if (in the latter case) only to the extent that such a consideration is necessary to weigh against one’s immediate and long-term self-interest when negotiating a contract (to say nothing of avoiding the free-riding problem) [7]. As pointed out by an anonymous reviewer, Kant also suggested when treating the Doctrine of Virtue in his Metaphysics of Morals [8], that the happiness of others is an obligatory rational end. Exploring this minimalist assumption in detail is an interesting project in itself, but outside the scope of this paper.

2 By other-regarding motivation, I simply mean a motivation to pay attention to the welfare of someone other than oneself.

3 I here rely on the subdivision of empathy into cognitive and emotional, or higher-level and lower-level, empathy, proposed by for example Goldman [21] and Shamay-Tzoory et al [22].

4 There is indeed suspicion that these effects are the result of publication bias, but the issue has not yet been settled.

5 A stringent utilitarian might well argue that any *extra* effort spent on increasing the welfare of close and loved ones is fundamentally immoral. That point is well taken. However, a moral enhancement intervention that made parents less likely to care for their children would probably not be well-received by the public, and such a stringent version of utilitarianism is hardly uncontroversial even among ethicists (for a perhaps extreme opposing point of view, see Asma [29]).

6 The converse of this is of course that increasing *s* will have the opposite (antisocial) effect. A closely related issue is that of self-esteem (see Roache [30] for an excellent overview). As noted by Baumeister et al [31]: “People high in self-esteem or narcissism are prone to bully others, to retaliate aggressively, and to be prejudiced against out-group members. … They may be willing to cheat and perform other antisocial, self-serving acts” (p. 37). (Thanks to Anders Sandberg for pointing this out to me).

7 The nature of the self is an essential topic in the Buddhist philosophy that underlies modern versions of mindfulness meditation. Though old and diverse, these teachings argue that the perception of a unified “self” is an illusion and that this illusion can be overcome through the practice of meditation.

8 There are also several studies looking at a particular form of mindfulness involving compassion training [38, 39]. Since prosocial effects of this type of training are more likely to arise from increases in compassion and empathy rather than decreases in sense of self, these studies are not relevant to the current case.

9 In the context of psychedelic research, this is often instead referred to as “ego-dissolution”, that is, a loss of sense of self as a separate entity from the rest of the world.

10 This, of course, is complicated by the wide range of definitions of empathy. Dolder et al [43] found that LSD acutely impaired recognition of negative emotions (which would qualify as a form of affective empathy under the definition used here), but also that subjects reported higher levels of “feeling *for*” others. The latter is interpreted as increases in emotional empathy in the study, but is more aligned with what is meant by other-regard in the model outlined in this paper (as opposed to the isomorphic “feeling *like*” others).

11 Their argument is admittedly more complex than that, but as a general description it will do.

12 Consider, for example, the case of actual antisocial preferences – that is, essentially negative values of *n* or *w* (although the model, as it is formulated here, does not deal with this type of deviation). For individuals with such preferences, it is clear that increases in the sum component *G* leads to even worse antisocial behavior. This underscores the contingency of the herein hypothesized moral effects of cognitive enhancement on, even small, preexisting prosocial motivations.

13 It is worth noting that added complexity in terms of a more varied taxonomy of motivations would actually *strengthen* this argument, since the possible ways in which meddling with them can then backfire only multiplies.
